# No clear relationship between antihypertensive class and cognitive function over 12 months in a cohort study of community-dwelling adults aged 80 and over

**DOI:** 10.1177/2040622318820849

**Published:** 2019-01-31

**Authors:** Ruth Peters

**Affiliations:** School of Public Health, Imperial College, London, UK; Neuroscience Research Australia, University of New South Wales, Sydney, Australia

**Keywords:** aged 80 and over, anticholinergic, antihypertensives, cognitive function

## Abstract

**Background:**

Hypertension is prevalent in older adults. Hypertension has also been associated with an increased risk of cognitive decline. However, evidence relating to the impact of antihypertensive use is mixed. Calcium-channel blockers (CCB) have been suggested as the most beneficial class of antihypertensive for protection of cognition in older adults, however, to date, there have been no cohort studies designed to examine this.

**Methods:**

Community-dwelling treated hypertensive adults aged 80 and over were recruited from general practice sites and followed for 1 year. Cognitive function was assessed at baseline and 12 months using the modified Mini-Mental State Exam (3MS). Regression was used to examine the association between 12-month exposure to antihypertensive class and change in cognitive function.

**Results:**

A total of 292 participants completed the study. Mean change in 3MS score was a rise of 0.53 [standard deviation (SD) 4.7] 3MS points in those taking CCBs (*n* = 135) compared with a drop of 0.09 (SD 5.1) in those without (*n* = 157) *p* = 0.28. There was no relationship between CCBs or between any antihypertensive class and change in cognitive function over 1 year. Additional analyses using a clinically meaningful fall of 5 or more 3MS points showed similar results.

**Conclusion:**

In a hypertensive community-dwelling older adult population treated with antihypertensives, there was no evidence that CCBs were protective of cognitive function over a 12-month exposure. If a protective effect is present, it may be small or require a longer treatment period. Larger longer studies are required for confirmation.

## Introduction

High blood pressure (BP) is associated with an increased risk of dementia or cognitive decline.^1^ Older adults, those aged 80 and over, are at high risk of having both high BP and of developing cognitive decline and are a fast growing sector of the population.^2,3^ BP lowering may reduce risk of cognitive decline and dementia,^4^ however meta-analyses report only small reductions in risk without statistical significance and data from placebo-controlled trials are sparse.^5^ Only one trial has shown a significant cognitive benefit with antihypertensive treatment in a hypertensive population, the Systolic Hypertension in Europe (Syst-Eur) trial.^6,7^ Syst-Eur was the only trial to use a calcium-channel blocker (CCB)-based antihypertensive-treatment regimen and reported a 50% reduction in incident dementia cases (Alzheimer’s disease and vascular dementia) in those ⩾60 years over a mean follow up of 2 years.^6,7^ It has been suggested that the Syst-Eur finding may be explained by various nonblood-pressure-lowering CCB-specific mechanisms including, but not limited to, reduced alteration in calcium homeostasis and improved amyloid-beta clearance.^8–10^ Despite this, the few observational data on CCB use in older adults show mixed results. The Canadian Study of Health and Ageing reported an increased risk of cognitive decline with CCB use [mean age 77.8 (SD5.7)],^11^ however the Leiden 85+ study reported CCBs were associated with significantly decelerated annual cognitive decline.^12^ Treatment for hypertension is recommended for those aged 80 and over^13^ and calcium-channel blockers alongside other classes of antihypertensive are now used routinely to lower BP in this group. Despite this, there have been no longitudinal cohort studies designed to evaluate the impact of CCBs, alone or in combination with other antihypertensive treatment, and impact, positive or negative, on cognitive function in this high-risk group. The Assessing the Impact of CCBs on COGnitive function in the very elderly (AI-COG) study aimed to remedy this by investigating the relationship between antihypertensive treatment and cognitive change over a 1-year period in a population based, older adult, general practice (GP)-based cohort study.

## Methods

Adults aged ⩾ 80 years were recruited from six GP sites across the northeast and northwest of England. Participants were community-dwelling older adults aged ⩾ 80 years and were required to be free from terminal disease likely to limit life to less than 12 months, to have a diagnosis of hypertension without a diagnosis of dementia and a Mini-Mental State Exam (MMSE) score of ⩾25. Participants attended their GP site for an initial assessment (baseline) and a follow-up assessment after 12 months (follow up). Reasonable transport costs were provided. Trained practice-based research nurses assessed participants and collected data from practice records. Information was collected at baseline and follow up and included current diagnoses, concomitant medications, height, weight, systolic and diastolic BP, consumption of alcohol, smoking behaviour, educational attainment and cognitive function. Antihypertensive medication was considered as present if it was from one of the main classes, CCBs, diuretics, beta blockers (BBs), angiotensin-converting enzyme (ACE) inhibitors or angiotensin receptor blockers (ARBs) and was prescribed at baseline and 12 months. Weight (kg) divided by height (m)^2^ was used to calculate body mass index (BMI). Educational attainment was classified as lower level, that is, primary and or secondary education, (essentially to age ~15 years in this population), compared with those with higher or further education. The modified MMSE, the 3MS, was used to assess cognitive function. The 3MS has fewer ceiling effects than the more widely used MMSE, is sensitive to cognitive decline, can be performed in a GP setting without placing undue burden on trial participants and gives rise to both MMSE and full 3MS scores.^14^ Permission to use the 3MS was provided by its developers. The collection of data on comorbidities from various domains (e.g. baseline reported pain, hearing loss, glaucoma, cataract, hernia, rheumatoid and osteoarthritis, osteoporosis, cholecystitis, kidney disease, atrial fibrillation, diabetes, heart failure, stroke, myocardial infarction, anxiety, depression, etc.) and concomitant medication also allowed the calculation of a baseline Frailty Index (FI). The FI is a measure of frailty or biological age and is calculated as the sum of a series of deficits [where these are binary, health related (e.g. presence of reported pain, hearing loss, obesity, etc.) and cover a variety of health areas] divided by the total number of variables used.^15^

Limited data on annual rates of cognitive change are available in those aged 80 and over treated with antihypertensives. A power calculation used annual mean change on the MMSE estimated from unpublished 12-month MMSE change data from participants aged 80 and over in the actively treated arms of the HYpertension in the Very Elderly Trial (HYVET), the Syst-Eur trial and published data from the Leiden 85+ study.^6,7,12,16^ An MMSE drop of 1.13 MMSE points [standard deviation (SD) 2.5] was estimated in the group not taking CCBs and a drop of 0.3 (SD 2.5) for the CCB-treated group. For 80% power at the 5% significance level, this resulted in a sample size of 143 in each group. Recruitment targets were set at 340 to account for an expected 20% attrition rate based on prior experience in this age group.^16^ Prior work with the participating GP sites established that around half of hypertensive older adults were taking CCBs. GP sites provided details of participant death or withdrawal occurring during the study follow up. Student’s *t* tests, Wilcoxon tests and chi-squared tests were used as appropriate for normally and non-normally distributed continuous and categorical variables to compare the baseline characteristics of those who remained in the study with those who left prior to their follow-up visit.

Cross-sectional analyses using multiple linear regression models were used to examine the baseline relationship between different antihypertensive classes and 3MS score adjusted for key risk factors [systolic and diastolic BP, BMI, diagnosis of diabetes or previous cardiovascular disease (stroke, heart failure, myocardial infarction, ischaemic heart disease)] selected for their potential to influence cognitive function. All analyses were additionally adjusted for age, sex, and education. Examination of BMI revealed a linear relationship with cognitive function; therefore, BMI was included as a continuous variable. Analyses were rerun with further adjustment for wider potential risk/protective factors, including prescribed statins, atrial fibrillation, number of medications with medium or high anticholinergic properties as defined in Salahudeen et al.^17^ or polypharmacy defined as five or more medications. Residuals were plotted as appropriate and the fit examined.

In longitudinal analyses, multiple linear regression was used to examine the influence of antihypertensive class on change in cognitive score with the follow-up 3MS score as the dependent variable adjusted for baseline 3MS score, key dementia risk factors, age and sex, as above. A similarly adjusted logistic regression was used to examine impact of antihypertensive class on a clinically meaningful drop in the 3MS (5 or more points).^18^

## Sensitivity analyses

To account for biological age, analyses were also run adjusted for the baseline frailty level using the FI, sex, and education. To examine the impact of exposure to a single class of antihypertensive, analyses were rerun, excluding all those prescribed more than one class.

## Impact of attrition

Logistic regression was used to examine any association between baseline characteristics and death or withdrawal. Furthermore, inverse probability weighting using age, sex, education, and baseline 3MS score was used to account for the possibility that those who left the study before completing the follow-up visit may have biased results by their withdrawal.

Ethical approval was obtained prior to study start and participants provided signed informed consent to participate in the study and to allow access to clinical records for up to 5 years for later collection of data on medication use and incident diagnoses of cognitive impairment or dementia. The results for the 12-month follow up are reported here. Data collection was *via* a password-protected remote data capture electronic-case-report-form system with inbuilt audit trails. Ethical approval was granted by the NRES Committee London, with the City Road and Hampstead reference number 13/LO/0635 [ClinicalTrials.gov identifier: NCT01868165].

## Results

**Figure 1.**
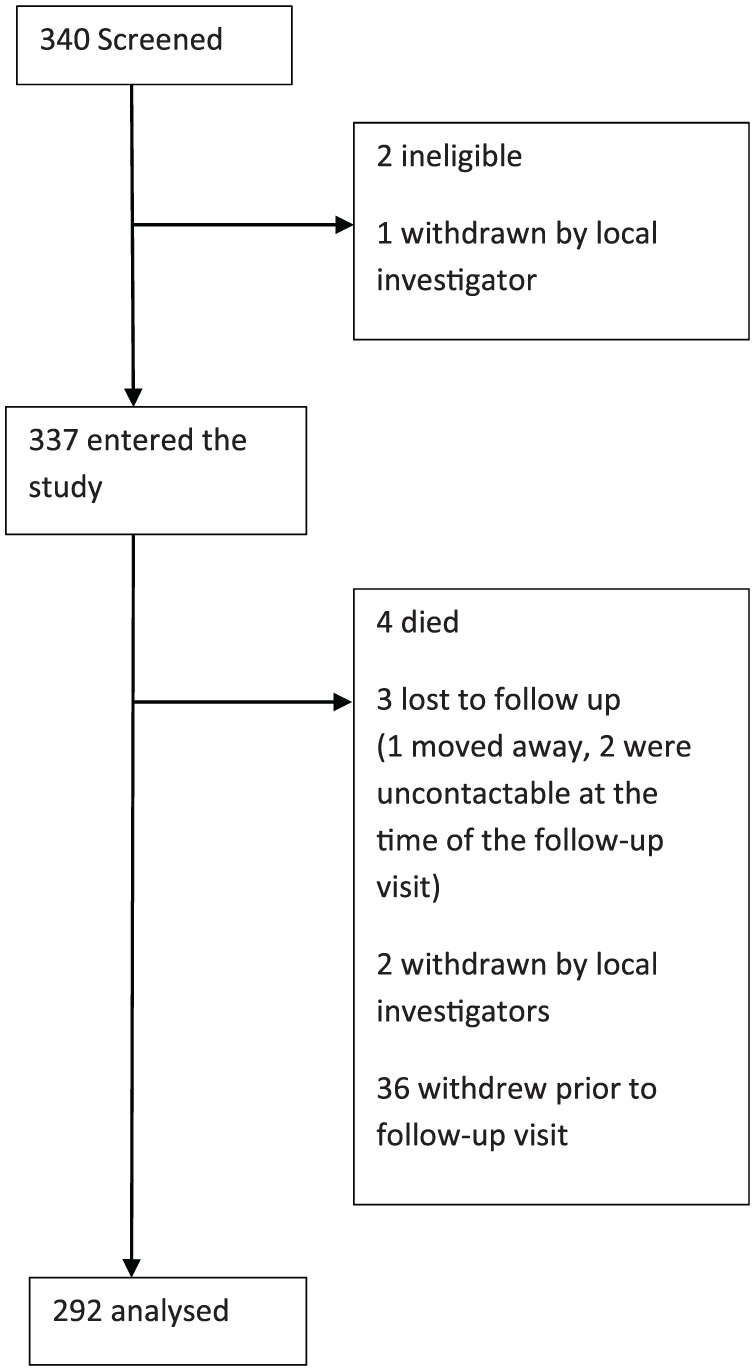
Flow chart of study participants.

A total of 340 community-dwelling older adults were screened, two were found to be ineligible and one was withdrawn by the local investigator prior to study entry. The remaining 337 participants completed a baseline visit. During the 12-month follow up, four participants died, three were lost to follow up, one of whom moved away and the other two were uncontactable at the time of the follow-up visit. Local investigators withdrew two participants and a further 36 participants chose to withdraw themselves. This left 292 with both baseline and follow-up visit data to be analysed. See flow chart in Figure 1. Table 1 shows baseline characteristics. Those who completed the study were significantly younger, more likely to be male, to have higher BMIs and higher cognitive scores. Missing data was less than 1%.

**Table 1.** Baseline characteristics.

Baseline characteristics, mean (standard deviation) or *n* (%)	Participants with 12-month follow up, *n* = 292	Participants without 12-month follow up, *n* = 45	
Age	83.5 (2.8)	84.8 (3.3)	*p* = 0.01
Female	161 (55.1)	32 (71.1)	*p* = 0.04
Systolic blood pressure baseline	137.1 (14.4)	136.1 (15.2)	*p* = 0.65
Diastolic blood pressure baseline	74.1 (9.1)	74.4 (8.1)	*p* = 0.81
Systolic blood pressure at follow up	138.7 (15.9)	N/A	N/A
Diastolic blood pressure at follow up	74.8 (9.7)	N/A	N/A
Number of recorded diagnoses	6.1 (3.6), median 6, interquartile range (IQR) 4–8	5.6 (3.7), median 5, IQR 3–7	*p* = 0.27
Cardiovascular disease^*^ present	81 (27.7)	7 (15.6)	*p* = 0.08
Diabetes	39 (13.4)	7 (15.6)	*p* = 0.69
Number of medications	9.3 (5.6), median 8, IQR 6–11	8.1 (3.9), median 2, IQR 2–3	*p* = 0.30
Number of antihypertensive medications	2.2 (0.9), median 2, IQR 2–3	2.3 (0.9), median 2, IQR 2–3	*p* = 0.51
Prescribed calcium-channel blockers	135 (46.2)	19 (42.2)	*p* = 0.62
Prescribed angiotensin receptor blockers	97 (33.2)	13 (28.9)	*p* = 0.56
Prescribed angiotensin-converting enzyme inhibitors	129 (44.2)	23 (51.1)	*p* = 0.38
Prescribed beta blockers	118 (40.4)	21 (46.7)	*p* = 0.43
Prescribed diuretics	145 (49.7)	26 (57.8)	*p* = 0.31
Prescribed statins	179 (61.3)	24 (53.3)	*p* = 0.31
Body mass index	27.5 (4.5)	26.0 (3.9)	*p* = 0.05
Current smoker	6 (2.1)	2 (4.4)	*p* = 0.33
Ex-smoker	125 (43.7)	19 (44.2)	*p* = 0.9
Units of alcohol per week	4.1 (6.2), median 1, IQR 0–6	4.4 (7.3), median 1, IQR 0–6	*p* = 0.91
Mini-Mental State Exam score	28.2 (1.4), median 29, IQR 27–29	27.6 (1.7), median 28, IQR 26–29	*p* = 0.04
Extended Mini-Mental State Exam score	93.6 (5.4), median 95, IQR 91–98	90.3 (6.9), median 92, IQR 85–95	*p* = 0.001
Frailty Index	0.21 (0.1), median 0.20, IQR 0.14–0.29	0.19 (0.1), median 0.17, IQR 0.11–0.26	*p* = 0.07

* Any diagnosis: stroke, heart failure, myocardial infarction or ischaemic heart disease.

Study participants had a mean age of 83.2 (SD 2.8) years, range 80.1–94.1 years. The majority, 55.1%, were female and reported white British ethnicity (98.6%) with the remainder reporting Asian (*n* = 1), Black (*n* = 1) and other unspecified ethnic groups (*n* = 2). The median number of diagnoses was 6 (mean 6.1) with the median number of medications 8 (mean 9.3). Overall mean BMI was 27.5 (4.5) with just 6 classified as underweight, 125 as overweight and 77 as obese. Very few participants smoked, although 43.7% were ex-smokers. Six participants completed primary education only; 195 reached secondary education and 90 achieved further education. Data on education was missing for one participant. Cognitive function 3MS score was high [mean 93.6 (SD 5.4); median 95] as expected in a community-dwelling population without cognitive decline. The population showed an average frailty level for their age group but did not include the very frail (median FI = 0.2, maximum FI = 0.49).

There were 135 participants taking CCBs, 145 taking diuretic-class antihypertensives, 129 taking ACE inhibitors, 118 taking BBs, and 97 taking ARBs. For those taking a single antihypertensive drug (*n* = 68), ARBs were the most common choice (*n* = 19) followed by CCBs (*n* = 17) and ACE inhibitors (*n* = 17). For dual therapy (*n* = 128), a diuretic plus an ACE inhibitor was the most common combination (*n* = 28) followed by a BB–ARB combination (*n* = 15). A total of 72 participants were taking three classes of antihypertensive, most commonly, diuretic, BB, ACE inhibitor or diuretic, CCB, or ACE inhibitor combinations (both *n* = 13) and 23 took four classes. Eight participants changed their antihypertensive medication class during the study; two men and six women. None of the eight were without antihypertensive treatment at study end. Reasons for change were not provided. Participants taking CCBs were more likely to have prior cardiovascular disease (34.1% compared with 22.3%, *p* = 0.03), had higher baseline systolic BP [mean values 139.2 mmHg (SD 13.5) compared with 135.4 (15.0), *p* = 0.03] and lower follow-up diastolic pressure [73.2 (9.7) compared with 76.1 (9.5), *p* = 0.01] see Supplementary Table 1.

## Cross-sectional analyses

At baseline; older age was associated with a lower 3MS score and worse cognitive function (β −0.34 [95% confidence intervals (CI) −0.56 to 0.11], whereas having a higher level of education was associated with higher cognitive function [β 2.04 (95% CI 0.68–3.40)]. There were no statistically significant relationships between antihypertensive class and cognitive function; see Table 2 for details.

**Table 2.** Associations between baseline variables and worsening cognition over 12 months.

	Cross-sectional analysis	Longitudinal analysis	
	Baseline 3MS, slope and 95% CI	12 month 3MS, slope and 95% CI	Fall of 5 or more 3MS points, OR and 95% CI
3MS baseline		0.72 (−0.62 to 0.83)	0.97 (0.91–1.03)
Education beyond secondary school	2.04 (0.68–3.40)	0.71 (−0.53 to 1.94)	0.38 (0.14–1.00)
Calcium-channel blockers	0.20 (−1.12 to 1.52)	0.58 (−0.60 to 1.77)	0.94 (0.42–2.10)
Diuretics	−0.30 (−1.50 to 0.95)	0.04 (−1.07 to 1.15)	0.65 (0.31–1.40)
Beta blockers	0.05 (−0.21 to 1.31)	−0.30 (−1.42 to 0.82)	1.44 (0.68–3.07)
Angiotensin-converting enzyme inhibitors	0.64 (−0.86 to 2.15)	−0.01 (−1.36 to 1.34)	0.82 (0.34–1.94)
Angiotensin receptor blockers	1.61 (−0.01 to 3.24)	0.05 (−1.42 to 1.51)	0.56 (0.21–1.49)
Body mass index baseline	−0.04 (−0.18 to 0.11)	0.03 (−0.10 to 0.16)	1.00 (0.92–1.09)
Systolic blood pressure (baseline)	0.01 (−0.04 to 0.06)	−0.02 (−0.06 to 0.03)	1.03 (1.00–1.06)
Diastolic blood pressure (baseline)	0.01 (−0.04 to 0.06)	−0.03 (−0.09 to 0.04)	1.05 (1.00–1.10)
Cardiovascular disease at baseline	−0.48 (−1.87 to 0.91)	0.15 (−1.09 to 1.39)	0.71 (0.30–1.69)
Diabetes	−1.72 (−3.55 to 0.11)	0.89 (−0.75 to 2.54)	1.46 (0.50–4.26)

* Adjusted for all covariates listed in the table plus age and sex.

3MS, modified Mini-Mental State Exam; CI, confidence interval; OR, odds ratio.

## Longitudinal analyses

The overall mean change in the MMSE was a drop of 0.03 points (SD 1.7), 0.01 (1.6) in the group taking CCBs and 0.04 (1.8) in the group without (*t* test *p* = 0.91; Table 3). For the 3MS, overall, the change was a rise of 0.20 (4.9), overall, and a rise of 0.53 (4.7) with CCBs and a drop of 0.09 (5.1) without (*t* test = 0.28; Table 3). Comparing each class of antihypertensive with the rest of the sample in turn showed similar results (Table 3).

**Table 3.** Antihypertensive drug classes and cognitive change over 12 months.

Antihypertensive class		MMSE 12-month change, mean (SD)	*t* test	3MS 12-month change, mean (SD)	*t* test
Calcium-channel blockers	Prescribed *n* = 135	−0.01 (1.6)	*p* = 0.91	0.53 (4.7)	*p* = 0.28
	Not prescribed *n* = 157	−0.04 (1.8)		−0.09 (5.1)	
Diuretics	Prescribed *n* = 145	0.01 (1.6)	*p* = 0.68	0.29 (4.9)	*p* = 0.75
	Not prescribed *n* = 147	−0.01 (1.9)		0.11 (4.9)	
Angiotensin-converting enzyme inhibitors	Prescribed *n* = 129	0.05 (1.8)	*p* = 0.47	0.18 (5.2)	*p* = 0.95
	Not prescribed *n* = 163	−0.09 (1.7)		0.21 (4.7)	
Angiotensin receptor blockers	Prescribed *n* = 97	−0.24 (1.8)	*p* = 0.12	−0.04 (4.3)	*p* = 0.53
	Not prescribed *n* = 195	0.08 (1.7)		0.31 (5.2)	
Beta blockers	Prescribed *n* = 118	−0.01 (1.8)	*p* = 0.88	−0.03 (4.6)	*p* = 0.64
	Not prescribed *n* = 174	−0.04 (1.6)		0.31 (5.1)	

MMSE, Mini-Mental State Exam; 3MS, modified Mini-Mental State Exam.

Multivariate linear regression examining the relationship between CCB use and 12-month 3MS score adjusted solely for baseline 3MS and rerun with additional adjustment for age, sex, education, BMI, systolic and diastolic BP, diabetes and prior cardiovascular disease reported slopes for CCB use of β 0.69 (95% CI −0.39 to 1.77) and β 0.60 (95% CI −0.52 to 1.72), respectively. Only baseline 3MS score was associated with later cognitive function such that a higher baseline score was associated with a higher score at follow up, β 0.74 (95% CI 0.64–0.84). When all antihypertensive classes were added into the model, there were no statistically significant relationships between class and cognitive change over 12 months (Table 2).

Logistic regression with a clinically meaningful change in 3MS, as the outcome showed similar results, and also finding a relationship between BP and increased risk of cognitive change, such that higher pressure was associated with increased risk (Table 2). Overall, the relationship between the antihypertensive classes, diuretic, ACE inhibitor, ARB and CCB was in the general direction of better cognitive function over time, whereas BBs showed the opposite pattern; however, cognitive change was small, and the relationships did not reach significance. Rerunning the analyses with additional adjustment for polypharmacy (defined as more than four current medications; Supplementary Table 2) or comparing only those with an exposure to a single antihypertensive class or running the analyses adjusted for frailty level, sex, and education (Supplementary Table 3) further attenuated the results. Similarly, inverse probability weighting did not materially change the results. FI alone was not significantly associated with either categorical or continuous measures of cognitive decline. There were no relationships between participant characteristics or antihypertensive drug class and risk of drop out or death during the study.

## Discussion

In a hypertensive community-dwelling older adult population followed for 12 months there were no statistically significant relationships between antihypertensive class and cognitive function at baseline or on cognitive change over time.

The direction of the results, that is, in a potentially positive direction for ACE inhibitor, CCB, ARB or diuretic and a more negative direction for BB is similar to, but not as strong as, that seen in the Newcastle 85+ study^19^ in a slightly older population. These results, taken together with the wider literature, support the role of BP lowering as unlikely to raise risk of cognitive impairment and possibly as having a potential role in lowering lower risk at least for ACE inhibitors, CCB, ARB and diuretics in older adults with hypertension, although caution should be applied, as additional adjustment further attenuated the potential relationships. The results also reinforce high BP as a potential risk factor in this group. The study demonstrates the relative stability of cognitive function in this group over the short term, finding only minimal change over a 12-month period. This was substantially lower than the change that was anticipated based on data from the HYVET, the Syst-Eur trial and the Leiden 85+ study. This may be because both the HYVET and the Syst-Eur trial were placebo-controlled trials evaluating the efficacy of antihypertensive treatment and as such, may have recruited a much more treatment naïve or certainly less aggressively treated population than current UK older adults where an emphasis is placed on hypertension and BP control. The Leiden 85+ study was a cohort study but did include participants slightly older than AI-COG and may have included those with some degree of existing impairment, both of which may have resulted in a faster fall in cognitive function. Further limitations come from the length of follow up. Although the Syst-Eur results were seen after a mean follow up of only 2.0 years, it has been suggested that a minimum of 5-year follow up is required.^20^ Nevertheless, the results presented here provide a valuable overview of an annual cognitive change in this population and as such, will inform the design of future studies. As a cohort study, the allocation of antihypertensive class was as currently practised at the sites; inevitably this means that many participants were exposed to more than one class. As the potential CCB effect was thought to be in addition to BP lowering, it should theoretically have been evident regardless, although arguably, the higher cardiovascular risk profile of those taking CCBs may have acted to mitigate potential benefits. Furthermore, additional analyses comparing only those who were taking single classes of antihypertensive did not materially change the results. Finally, a detailed neuropsychological battery would have been a more sensitive tool to assess cognitive function; however, it would have been difficult to execute on site. The use of the 3MS was more robust than more widely used and shorter screening tools, facilitated the delivery of the study in busy GP sites and did not place undue burden on the participants; however, it may still have been subject to ceiling effects. For example, although cognitive function might be expected to be related to frailty (even in an older adult group without overt frailty) there was no relationship between baseline 3MS and baseline FI score.

Overall, the study provides a robust description of a community-dwelling older adult hypertensive population. It supports the concept of antihypertensives as maybe having the potential to lower risk and as unlikely to increase risk of cognitive decline, and reinforces the role of high BP as increasing risk in this group. Further longer-term studies are required to evaluate the role, if any, of different antihypertensive classes, or specific antihypertensive drugs and the impact of cognitive function.^21,22^

## Supplemental Material

Baseline_characteristics – Supplemental material for No clear relationship between antihypertensive class and cognitive function over 12 months in a cohort study of community-dwelling adults aged 80 and over Click here for additional data file. Supplemental material, Baseline_characteristics for No clear relationship between antihypertensive class and cognitive function over 12 months in a cohort study of community-dwelling adults aged 80 and over by Ruth Peters in Therapeutic Advances in Chronic Disease

## References

[1] Skoog I Lernfelt B Landahl S et al 15 year longitudinal study of blood pressure and dementia. Lancet 1996; 347: 1141–1145. 8609748 10.1016/s0140-6736(96)90608-x

[2] Health Survey for England. 2009 trend tables. UK: Health and Social Care Information Centre, https://data.gov.uk/dataset/health_survey_for_england (2010; accessed 4 June 2016).

[3] United Nations, Department of Economic and Social Affairs, Population Division (2017). World Population Ageing 2017 (ST/ESA/SER.A/408). http://www.un.org/en/development/desa/population/publications/pdf/ageing/WPA2017_Report.pdf (accessed 18 Dec 2018)

[4] Gelber R Ross G Petrovitch H et al Antihypertensive medication use and risk of cognitive impairment. The Honolulu-Asia aging study. Neurol 2013; 81: 1–8. 10.1212/WNL.0b013e3182a351d4 PMC3885214 23911753

[5] Prince M Wimo A Guerchet M et al World Alzheimer report 2015, the global impact of dementia. London: Alzheimer’s Disease International, 2015.

[6] Forette F Seux M Staessen J et al.; Systolic Hypertension in Europe Investigators. The prevention of dementia with antihypertensive treatment. Arch Int Med 2002; 162: 2046–2052. 12374512 10.1001/archinte.162.18.2046

[7] Forette F Seux M Staessen J et al Prevention of dementia in a randomised double blind placebo controlled systolic hypertension in Europe (Syst-Eur) trial. Lancet 1998; 352: 1347–1351. 9802273 10.1016/s0140-6736(98)03086-4

[8] Paris D Bachmeier C Patel N et al Selective antihypertensive dihydropyridines lower Aß accumulation by targeting both the production and the clearance of Aß across the blood-brain barrier. Molecul Med 2011; 17:149–162. 10.2119/molmed.2010.00180 PMC3060987 21170472

[9] Lovell MA Abner E Kryscio R et al Calcium channel blockers, progression to dementia, and effects on amyloid beta peptide production. Oxid Med Cell Longev 2015; 2015: 787805. 26221415 10.1155/2015/787805 PMC4499419

[10] Ros Agabiti-Rosei E Heagerty AM Rizzoni D Effects of antihypertensive treatment on small artery remodelling. J Hypertens 2009; 27: 1107–1114. 19293726 10.1097/HJH.0b013e328329272e

[11] Maxwell C Hogan D Ebly E. Calcium-channel blockers and cognitive function in elderly people: results from the Canadian study of health and aging. CMAJ 1999; 161: 501–506. 10497605 PMC1230578

[12] Trompet S Westendorp R Kamper A et al Use of calcium antagonists and cognitive decline in old age The Leiden 85-plus study. Neurobiol Aging 2008; 29: 306–308. 17101196 10.1016/j.neurobiolaging.2006.10.006

[13] Mancia G Laurent S Agabiti-Rosei E et al Reappraisal of European guidelines on hypertension management: a European Society of Hypertension Task Force document. J Hypertens 2009; 27: 2121–2158. 19838131 10.1097/HJH.0b013e328333146d

[14] Teng EL Chui HC. The Modified Mini-Mental State (3MS) examination. J Clin Psychiatr 1987; 48: 314–318. 3611032

[15] Searle SD Mitnitski A Gahbauer EA et al A standard procedure for creating a frailty index. BMC Geriatr 2008; 8: 24. 18826625 10.1186/1471-2318-8-24 PMC2573877

[16] Peters R Beckett N Forette F et al.; HYVET Investigators. Incident dementia and blood pressure lowering in the hypertension in the very elderly trial cognitive function assessment (HYVET-COG): a double-blind, placebo controlled trial. Lancet Neurol 2008; 7: 683–689. 18614402 10.1016/S1474-4422(08)70143-1

[17] Salahudeen M Duffull S Nishtala P. Anticholinergic burden quantified by anticholinergic risk scales and adverse outcomes in older people: a systematic review. BMC Geriatr 2015; 15: 31. 25879993 10.1186/s12877-015-0029-9 PMC4377853

[18] Andrew MK Rockwood K. A five-point change in modified mini-mental state examination was clinically meaningful in community-dwelling elderly people. J Clin Epidemiol 2008; 61: 827–831. 18468855 10.1016/j.jclinepi.2007.10.022

[19] Peters R Collerton J Granic A et al Antihypertensive drug use and risk of cognitive decline in the very old: observational study - the Newcastle 85+ Study. J Hypertens 2015; 33: 2156–2164. 26237554 10.1097/HJH.0000000000000653

[20] Skoog I. Antihypertensive treatment and dementia prevention. Lancet Neurol 2008; 7: 664–665. 18614403 10.1016/S1474-4422(08)70144-3

[21] Peters R Schuchman M Peters J et al Relationship between antihypertensive medications and cognitive impairment: part II. Review of physiology and animal studies. Curr Hypertens Rep 2016; 18: 66. 27492369 10.1007/s11906-016-0673-2 PMC4988998

[22] Yasar S Schuchman M Peters J et al Relationship between antihypertensive medications and cognitive impairment: part I. Review of human studies and clinical trials. Curr Hypertens Rep 2016; 18: 67. 27492370 10.1007/s11906-016-0674-1 PMC4975763

