# Supplementary material for: No clear relationship between antihypertensive class and cognitive function over 12 months in a cohort study of community-dwelling adults aged 80 and over
Source: Ther Adv Chronic Dis. 2019 Jan 31;10:2040622318820849. doi: 10.1177/2040622318820849 (PMC6357296; doi:10.1177/2040622318820849)
Supplement: Baseline_characteristics – Supplemental material for No clear relationship between antihypertensive class and cognitive function over 12 months in a cohort study of community-dwelling adults aged 80 and over [file Baseline_characteristics.pdf]

Supplementary table 1 Characteristics of those prescribed calcium channel blockers and those not prescribed calcium channel blockers

Baseline characteristics<br>Mean (Standard Deviation) or n (%)	Participants treated with CCB n=135	Participants not treated with CCB n=157	
Age	83.7 (2.9)	83.4 (2.8)	P=0.4950
Female	75 (55.5)	86 (54.8)	P=0.9066
Systolic blood pressure baseline	139.2 (13.5)	135.4 (15.0)	P=0.0254
Diastolic blood pressure baseline	73.6 (9.1)	74.4 (9.0)	P=0.4504
Systolic blood pressure at follow-up	138.8 (16.3)	138.5 (15.6)	P=0.8724
Diastolic blood pressure at follow-up	73.2 (9.7)	76.1 (9.5)	P=0.0111
Number of recorded diagnoses	5.9 (3.5)	6.3 (3.7)	P=0.3104
Cardiovascular disease present	46 (34.1)	35 (22.3)	P=0.0266
Diabetes	23 (17)	16 (10.2)	P=0.1197
Number of medications	9.5 (6.0)	9.0 (5.1)	P=0.4894
Prescribed statins	75 (55.6)	104 (66.2)	P=0.0710
Body Mass Index (BMI)	27.8 (4.4)	27.2 (4.6)	P=0.2600
Current smoker	3 (2.2)	3 (1.9)	P=1.000
Units of alcohol per week	4.7 (7.2)	3.7 (5.2)	P=0.1871

Mini-Mental State Exam score	28.2 (1.5)	28.2 (1.4)	P=0.9290
Extended Mini-Mental State Exam (3MS) score	93.6 (5.7)	93.7 (5.1)	P=0.9127
Frailty Index (FI)	0.21 (0.08)	0.21 (0.09)	P=0.8070

Supplementary Table 2 Sensitivity analyses with polypharmacy.

	Cross sectional analysis	Longitudinal analysis	
	Baseline 3MS†, slope and 95% Confidence Intervals (CI)	12 month 3MS†, slope and 95% CI	Fall of 5 or more 3MS† points, OR and 95% CI
3MS† baseline		0.73 (0.62: 0.84)	0.97 (0.91: 1.03)
Education beyond secondary school	1.91 (0.55:3.27)	0.77 (-0.46: 2.01)	0.37 (0.14: 1.00)
Calcium Channel Blockers (CCB)	0.33 (1.00:1.65)	0.51 (-0.68: 1.69)	0.95 (0.42: 2.12)
Diuretics	-0.15 (-1.40:1.10)	-0.05 (-1.17: 1.07)	0.66 (0.30: 1.43)
Beta Blockers	0.33 (-0.96:1.62)	-0.47 (-1.62: 0.69)	1.45 (0.68: 3.13)
Angiotensin Converting Enzyme inhibitors (ACE)	0.75 (-0.75:2.25)	-0.08 (-1.43: 1.27)	0.82 (0.35: 1.95)
Angiotensin Receptor Blockers (ARB)	1.85 (0.21:3.49)	-0.11 (-1.59: 1.38)	0.56 (0.21: 1.52)
Body Mass Index (BMI) baseline	-0.03 (-0.17:0.11)	0.03 (-0.10: 0.16)	1.00 (0.92: 1.10)
Systolic blood pressure (baseline)	0.01 (-0.04:0.06)	-0.02 (-0.06: 0.03)	1.03 (1.00: 1.06)
Diastolic blood pressure (baseline)	0.01 (-0.07:0.08)	-0.03 (-0.09: 0.04)	1.05 (1.00: 1.10)

Cardiovascular disease at baseline	-0.42 (-1.80:0.97)	0.12 (-1.13: 1.36)	0.72 (0.30: 1.70)
Diabetes	-1.59 (-3.42:0.24)	0.83 (-0.82: 2.48)	1.47 (0.50: 4.29)
Polypharmacy	-1.64 (-3.41:0.14)	0.98 (-0.62: 2.58)	1.09 (0.37: 3.17)
	Cross sectional analysis	Longitudinal analysis	
	Baseline 3MS†, slope and 95% Confidence Intervals (CI)	12 month 3MS†, slope and 95% CI	Fall of 5 or more 3MS† points, OR and 95% CI

†Modified Mini-Mental State Exam 3MS

*Adjusted for all covariates listed in the table plus age and sex.

Supplementary Table 3 Sensitivity analyses with frailty level.

	Cross sectional analysis	Longitudinal analysis	
	Baseline 3MS†, slope and 95% Confidence Intervals (CI)	12 month 3MS†, slope and 95% CI	Fall of 5 or more 3MS† points, OR and 95% CI
3MS† baseline		0.74 (0.63: 0.84)	0.97 (0.91: 1.03)
Education beyond secondary school	2.24 (0.89: 3.59)	0.77 (-0.44: 1.99)	0.41 (0.16: 1.06)
Calcium Channel Blockers (CCB)	-0.05 (-1.32: 1.23)	0.55 (-0.57: 1.68)	0.93 (0.44: 1.96)
Diuretics	-0.45 (-0.32: 1.23)	-0.10 (-1.20: 0.99)	0.65 (0.32: 1.35)
Beta Blockers	-0.07 (-0.34: 1.18)	-0.48 (-1.59: 0.64)	1.26 (0.61: 2.59)
Angiotensin Converting Enzyme inhibitors (ACE)	0.60 (-0.92: 2.11)	-0.08 (-1.41: 1.26)	0.81 (0.34: 1.90)
Angiotensin Receptor Blockers (ARB)	1.66 (0.00: 3.32)	-0.29 (-1.76: 1.18)	0.72 (0.27: 1.92)
[illegible]	[illegible]	[illegible])	0.99 (0.94: 1.03)

†Modified Mini-Mental State Exam 3MS

*Adjusted for all covariates listed in the table plus age and sex.
